# The non-canonical hydroxylase structure of YfcM reveals a metal ion-coordination motif required for EF-P hydroxylation

**DOI:** 10.1093/nar/gku898

**Published:** 2014-10-01

**Authors:** Kan Kobayashi, Assaf Katz, Andrei Rajkovic, Ryohei Ishii, Owen E. Branson, Michael A. Freitas, Ryuichiro Ishitani, Michael Ibba, Osamu Nureki

**Affiliations:** 1 Department of Biological Sciences, Graduate School of Science, The University of Tokyo, 2-11-16 Yayoi, Bunkyo-ku, Tokyo 113-0032, Japan; 2 Global Research Cluster, RIKEN, 2-1, Hirosawa, Wako, Saitama, 351-0198, Japan; 3 Department of Microbiology, Ohio State University, Columbus, OH 43210, USA; 4 Molecular, Cell, and Developmental Biology Program, Ohio State University, Columbus, OH 43210, USA; 5 Department of Biochemistry, Ohio State University, Columbus, OH 43210, USA; 6 Comprehensive Cancer Center, Ohio State University, Columbus, OH 43210, USA; 7 Department of Molecular Virology, Immunology and Medical Genetics, Ohio State University, Columbus, OH 43210, USA; 8 Ohio State Biochemistry Program, Center for RNA Biology, Ohio State University, Columbus, OH 43210, USA

## Abstract

EF-P is a bacterial tRNA-mimic protein, which accelerates the ribosome-catalyzed polymerization of poly-prolines. In *Escherichia coli*, EF-P is post-translationally modified on a conserved lysine residue. The post-translational modification is performed in a two-step reaction involving the addition of a β-lysine moiety and the subsequent hydroxylation, catalyzed by PoxA and YfcM, respectively. The β-lysine moiety was previously shown to enhance the rate of poly-proline synthesis, but the role of the hydroxylation is poorly understood. We solved the crystal structure of YfcM and performed functional analyses to determine the hydroxylation mechanism. In addition, YfcM appears to be structurally distinct from any other hydroxylase structures reported so far. The structure of YfcM is similar to that of the ribonuclease YbeY, even though they do not share sequence homology. Furthermore, YfcM has a metal ion-coordinating motif, similar to YbeY. The metal ion-coordinating motif of YfcM resembles a 2-His-1-carboxylate motif, which coordinates an Fe(II) ion and forms the catalytic site of non-heme iron enzymes. Our findings showed that the metal ion-coordinating motif of YfcM plays an essential role in the hydroxylation of the β-lysylated lysine residue of EF-P. Taken together, our results suggested the potential catalytic mechanism of hydroxylation by YfcM.

## INTRODUCTION

Protein synthesis by the ribosome is facilitated by elongation factors (EFs). During elongation, the translation machinery incorporates a chemically diverse set of amino acids with general kinetic uniformity. However, peptide bond formation rates have been shown *in vitro* to vary between individual amino acids (1,2). In particular, proline has a relatively weak propensity to react with aminoacyl-tRNA (or its analog puromycin) for the peptide bond formation (1–6). EF-P binds between the P and E sites of ribosomes and enhances the synthesis of poly-proline sequences by ribosomes (3–7). In the absence of EF-P, ribosomes stall on mRNAs encoding poly-proline sequences, and the abundance of poly-proline containing proteins decreases (3–5).

In order for EF-P to perform its function efficiently, a post-translational modification at the conserved residue Lys34 (*Escherichia coli* numbering) is required in *E. coli* and *Salmonella* Typhimurium (3,8–11) (Supplementary Figure S1). In EF-P, Lys34 structurally corresponds to the 3′ end of tRNA (12,13), and its modification requires the enzymes PoxA and YjeK (8). YjeK is an aminomutase that converts (*S*)-α-lysine to (*R*)-β-lysine, which is subsequently attached to Lys34 of EF-P by PoxA (8) (Supplementary Figure S1). PoxA is a paralog of lysyl-tRNA synthetase but lacks an anticodon-binding domain. PoxA β-lysylates Lys34 of EF-P, in a similar manner to the lysylation of the 3′-end of tRNA by lysyl-tRNA synthetase (14–16). Disruption of the β-lysylation modification pathway results in pleiotropic phenotypes, similar to the disruption of *efp* in *E. coli* and *Salmonella enterica* (4,11,17,18). Therefore, the β-lysylation of EF-P is essential for its function. The structural model of β-lysylated EF-P in complex with the ribosome suggested that Lys34 and the β-lysine moiety protrude into the peptidyl transferase center, where they contact and stabilize the 3′-CCA end of tRNA in the P site (8,19).

A recent study revealed that the β-lysylated Lys34 of EF-P is subsequently hydroxylated at C4(γ) or C5(δ) by YfcM (19) (Supplementary Figure S1). This modification does not affect the activity of β-lysylated EF-P in the synthesis of proteins containing poly-prolines (3), although it stimulates the reactivity with puromycin (18). However, a previous report suggested that the hydroxyl moiety at C5(δ), but not that at C4(γ), could contact tRNA in the P site and provide further stability (19). This possible role for hydroxylation in EF-P function is similar to a recent finding showing that translation termination efficiency is promoted by the hydroxylation of Lys63 (*human* numbering) in eukaryotic release factor 1 (eRF1) (20). Since Lys63 is considered to recognize the invariant uridine of stop codons (21), the added hydroxyl moiety could be involved in stabilizing this interaction (20). In a similar manner, the hydroxyl moiety of EF-P is speculated to play an auxiliary role, such as stabilizing the conformation of the tRNA in the P site, the rRNA or the β-lysine moiety attached to Lys34 of EF-P.

EF-P is a homolog of eukaryotic initiation factor 5A (eIF5A). Analogous to EF-P, a conserved lysine residue of eIF5A is modified with a hypusine residue, in a two-step reaction (22). The 4-amino butyl moiety of spermidine is attached to the ϵ-amino group of the lysine residue by deoxyhypusine synthase (DHS) and is subsequently hydroxylated by deoxyhypusine hydroxylase (DOHH). Based on the crystal structures of DHS, the mechanism of eIF5A deoxyhypusination is well understood (23,24), whereas the structure of DOHH has not been determined, except for one predicted model (25). Thus, although the first modification step has been well studied for both EF-P and eIF5A (β-lysylation in EF-P and deoxyhypusination in eIF5A), the subsequent hydroxylation mechanisms are not clear in either case.

Here, we present the crystal structure of YfcM from *E. coli*. Although YfcM is structurally different from any other hydroxylation enzymes reported so far, YfcM shares similarity with the ribonuclease YbeY, despite their low sequence homology (26). Furthermore, the three-histidine motif of YbeY for the coordination of a metal ion is replaced with a putative 2-His-1-carboxylate motif for the coordination of an Fe(II) in YfcM (26,27). Combined with functional analyses, our structure provides insights into the mechanism of Lys34 hydroxylation in β-lysylated EF-P.

## MATERIALS AND METHODS

### Sample preparation and crystallization

We expressed, purified and crystallized *E. coli* YfcM as previously described (28). Subsequently, we crystallized selenomethionine (SeMet)-labeled YfcM to calculate phases by the multi-wavelength anomalous dispersion (MAD) method. Since YfcM harbors only one methionine residue (Met143), except for Met1 (Supplementary Figure S2), the L107M mutation was introduced in order to amplify the anomalous dispersion signals of the selenium atoms (Supplementary Figure S2). The methionine-auxotrophic *E. coli* strain B834(DE3)CodonPlus (Novagen) was transformed with the plasmid expressing YfcM (L107M), and the cells were grown in Core medium (Wako), supplemented with 25 μg/ml kanamycin, 1% glucose, Vitamin Mixture (Sigma-Aldrich), 1 mM MgSO_4_, 15 μM FeSO_4_ and 50 μg/ml seleno-L-methionine. SeMet-labeled YfcM (L107M) was expressed and purified in the same manner as the native protein. The crystals of SeMet-labeled YfcM (L107M) were obtained under conditions similar to those used for the native protein.

### Data collection and structure determination

X-ray diffraction experiments were performed, as previously described (28). Diffraction data were collected at beamlines BL32XU and BL41XU at SPring-8 (Harima, Japan), and were processed with the HKL2000 program (HKL Research). The data collection statistics are provided in Supplementary Table S1. The structure of SeMet-labeled YfcM (L107M) was determined by the MAD method, using the selenium anomalous dispersion signals. Two selenium sites were detected in the asymmetric unit with the SHELXC and SHELXD programs (29). The refinement of the selenium sites and the phase calculations were performed with the SHARP program (30), followed by solvent flattening with the SOLOMON program (31). The generated electron density map revealed one molecule of YfcM in the asymmetric unit, which enabled automated model building with the RESOLVE program (32). The model was improved by manual model building with the COOT program (33). The improved model was used as the search model for molecular replacement against the native data set up to 1.45 Å resolution with the Molrep program (34). The native model was further improved by alternating cycles of model building with the COOT program and refinement with the Refmac (35) and PHENIX (36) programs. The native model was finally refined to an *R*_work_ of 14.8%, with an *R*_free_ of 17.5%. The phase calculation and structural refinement statistics are summarized in Supplementary Tables S1 and S2, respectively.

### Detection of YfcM-bound Fe ion by atomic absorption spectrometry

The purified native YfcM protein was dialyzed against dialysis buffer (50 mM Tris-HCl pH 7.0, 200 mM NaCl and 1 mM 2-mercaptoethanol) overnight at 4°C. An FeSO_4_ solution was added to the dialyzed product to a final concentration of 3 mM_._ After filtration, YfcM was purified by chromatography on a Superdex 75 10/300 column (GE Healthcare). The fraction containing YfcM was collected (0.86 mg/ml). The Fe content of this YfcM protein, together with that of the dialyzed product of apo YfcM (1.6 mg/ml), was analyzed by atomic absorption spectrometry (Supplementary Table S3). Samples were then submitted to the Trace Element Research Laboratory at Ohio State University. Quantitative measurements of Fe were performed on a Perkin-Elmer Optima 4300DV Inductively Coupled Plasma Optical Emission Spectrometer.

### Structure determination of Co(II)-bound YfcM

To confirm that an Fe(II) ion binds to the putative catalytic site of YfcM, we tried to bind a Co(II) ion to this site in an Fe(II)-mimicking manner and to detect it in the anomalous difference Fourier map. For the preparation of Co(II)-bound YfcM crystals, the native YfcM crystals were soaked for 3 min in a harvest solution, containing 3 mM CoCl_2_, 27% PEG 3350, 210 mM (NH_4_)_2_SO_4_ and 120 mM Bis-Tris, pH 5.5. The X-ray diffraction data were collected at the peak wavelength of the Co K-shell absorption edge (1.60490 Å) at beamline BL41XU at SPring-8 (Harima, Japan), and were processed with the HKL2000 program (HKL Research). The anomalous difference Fourier map of Co(II)-bound YfcM was calculated with the PHENIX program (36), using the native structure of YfcM and the dataset collected from the crystal of Co(II)-bound YfcM. The Co(II)-bound YfcM structure was refined in the same manner as the native YfcM structure, except that Refmac program was not used. The model was finally refined to an *R*_work_ of 17.0%, with an *R*_free_ of 20.7%. The data collection and structural refinement statistics are provided in Supplementary Table S4. The distance between Co(II) ion and each ligand atom is summarized in Supplementary Table S5. The structure factors and coordinates of YfcM have been deposited in the Protein Data Bank (accession code: 4PDN for the native form and 3WTR for the Co(II)-bound form).

### Preparation of EF-P for the *in vitro* hydroxylation assay

The gene encoding EF-P-His_6_ was cloned into the plasmid pET-33b(+). The *efp* gene was amplified using the primers ACCTAGCCATGGGCGCAACGTACTATAGCAACGAT and CTAGCACTCGAGCTTCACGCGAGAGACGTATTC. The PCR product was digested with the NcoI and XhoI enzymes and inserted at the corresponding restriction sites of the plasmid. EF-P was purified using standard metal chromatography protocols. Briefly, the protein was produced in *E. coli* BL21 (DE3) cells and lysed by sonication. The cell lysate was then loaded onto a Ni-NTA column. The column was washed with 50 volumes of a solution, containing 25 mM Tris-HCl pH 8.0, 300 mM NaCl, 10% glycerol (Buffer 1) and 5 mM imidazole. The column was further washed with 15 volumes of Buffer 1 with 20 mM imidazole, and finally the protein was eluted in Buffer 1 with 40 mM imidazole. The fractions containing the protein were pooled and dialyzed against a solution containing 50 mM Tris-HCl pH 8.0, 150 mM NaCl, 2 mM 2-mercaptoethanol and 50% glycerol.

### Aminoacylation of EF-P

Aminoacylation was performed under conditions similar to those reported previously (15). Briefly, the reaction was performed in a mixture containing 100 mM glycine KOH, pH 9.0, 30 mM KCl, 12 mM MgCl_2_, 3 mM 2-mercaptoethanol, 10 mM ATP, 300 μM (*R*)-β-lysine (gift from Dr. Craig Forsyth, Ohio State University, Columbus, OH, USA), 1 μM *E*. *coli* PoxA and 40 μM EF-P. After a 30 min incubation at 37°C, the mixture was diluted with one volume of Buffer 1 plus 5 mM imidazole and loaded onto a Ni-NTA Sepharose column. The column was washed with 100 volumes of Buffer 1 plus 5 mM imidazole, and eluted with Buffer 1 plus 300 mM imidazole. The fractions that contained the protein were collected and dialyzed against 50 mM Tris-HCl pH 8.0, 150 mM NaCl, 2 mM 2-mercaptoethanol and 50% glycerol. The protein was then stored at −80°C.

### *In vitro* hydroxylation of β-lysylated EF-P

The components required for hydroxylating (*R*)-β-lysyl-EF-P have not been defined, and the hydroxylation of (*R*)-β-lysyl-EF-P was achieved by the method described previously (19). (*R*)-β-lysyl-EF-P (5 μM) was hydroxylated using 5 μM of wild-type (WT) or YfcM variants in a complex reaction mixture, consisting of 5 mM 2-mercaptoethanol, 125 mM NaCl, 25 mM KCl, 10 μM FeSO_4_, 10 μM MgCl_2_, 10 μM ATP, 10 μM GTP, 10 μM Coenzyme A (Sigma Aldrich, USA), 10 μM FAD (Sigma Aldrich, USA), 10 μM PLP (Sigma Aldrich, USA), 10 μM oxaloacetic acid (Sigma Aldrich, USA), 10 μM thiamine, 10 μM reduced glutathione, 10 μM L-ascorbic acid (Sigma Aldrich, USA), 10 μM α-ketoglutarate, 5 mM NADPH and 5.8 A_280_ Absorbance Units of *E. coli* Δ*yfcm* 3.5kDa filtered extract. The hydroxylation dependence on iron was tested by adding 200 μM 2,2′-Bipyridine (Sigma Aldrich, USA) and removing 10 μM FeSO_4_ from the reaction**_._** The mixture was incubated for 155 min at 30°C. The reaction was stopped by precipitating the proteins with 20% TCA. The pellet was then washed twice with 80% ethanol and once with acetone. The sample was air dried and solubilized in ProteaseMax surfactant (Promega, USA). The sample was then digested with a Trypsin/LysC mix (Promega, USA) according to the manufacturer's protocol. The hydroxylated fraction was then analyzed by liquid chromatography coupled to mass spectrometry (Supplementary methods, Supplementary Figure S3 and Supplementary Tables S6 and S7).

## RESULTS

### Overall structure

**Figure 1.**
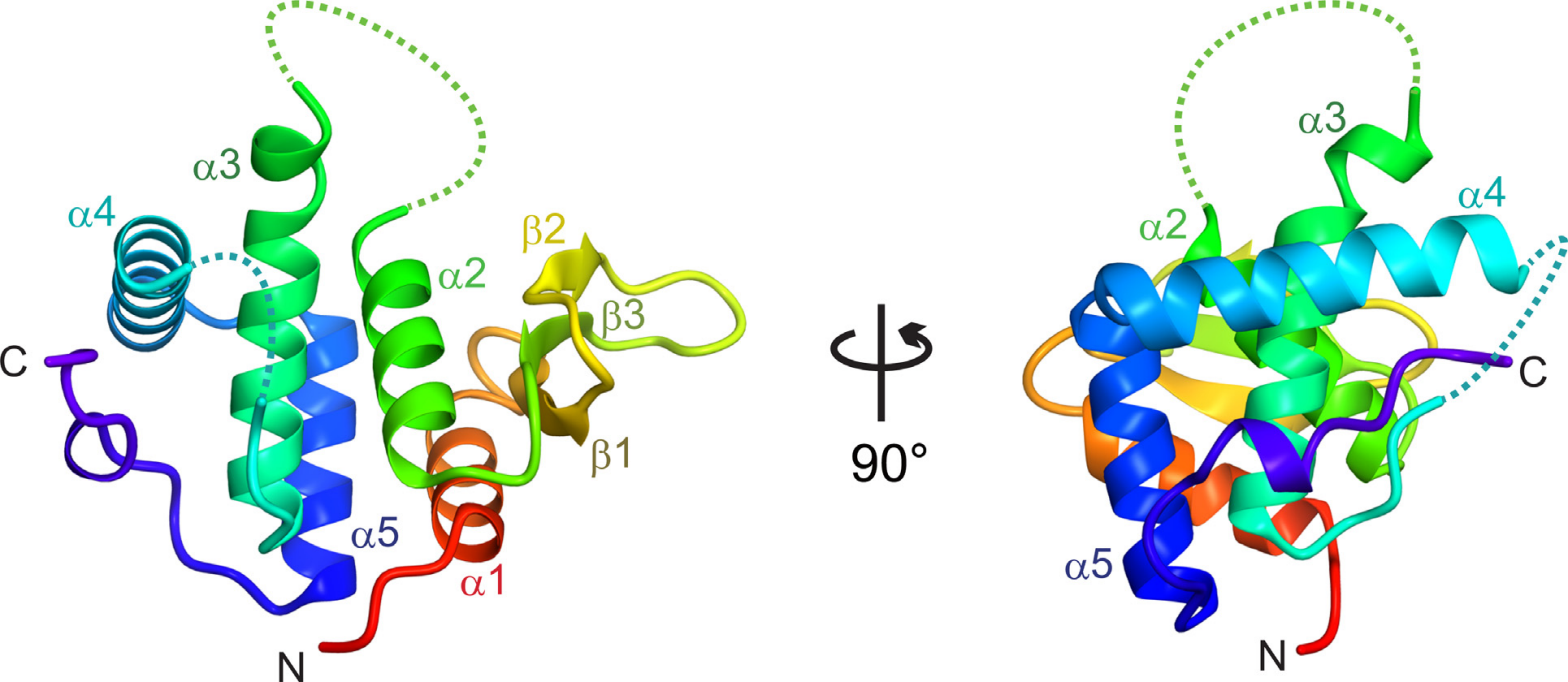
Overall structure of YfcM, seen from two perpendicular directions. The structure is depicted by a rainbow-colored ribbon model. Disordered loops are represented as dashed lines.

To reveal the mechanism of EF-P hydroxylation catalyzed by YfcM, the crystal structure of YfcM was determined at 1.45 Å resolution by the MAD method. In the crystal, YfcM exists as a monomer. The structure consists of five α-helices (α1–α5) and a β sheet composed of three β strands (β1–β3) (Figure 1). The residues forming the loop between α2 and α3 were removed by the trypsin digestion during the crystallization, as confirmed by N-terminal sequencing and MALDI-TOF mass spectrometry analyses (28). As a result, residues Gly68–Ser92 are not modeled because of the lack of electron density. In addition, the residues Met1-Asn2, Val118-Asp130 and Ala180 to the C-terminus are also structurally disordered.

### YfcM structurally resembles YbeY

In order to identify proteins with structures similar to that of YfcM, we performed a structural homology search using the PDBeFold server. The results showed that YfcM is structurally similar to the UPF0054 family proteins, such as ribonuclease YbeY from *E. coli* (PDB code: 1XM5) with a Q score of 0.14, and AQ_1354 from *Aquifex aeolicus* (PDB code: 1OZ9) with a Q score of 0.11, despite the low sequence similarity between the YfcM and UPF0054 family proteins (26,37). The structures of the UPF0054 family proteins reported so far consist of one four-stranded β sheet and five or six α-helices (26,37–39). Additionally, the UPF0054 family proteins are characterized by a highly conserved three-histidine motif for the coordination of a metal ion, postulated to be a nickel or zinc ion (26,37–41), which plays an essential role in the ribonuclease activity of YbeY (41).

**Figure 2.**
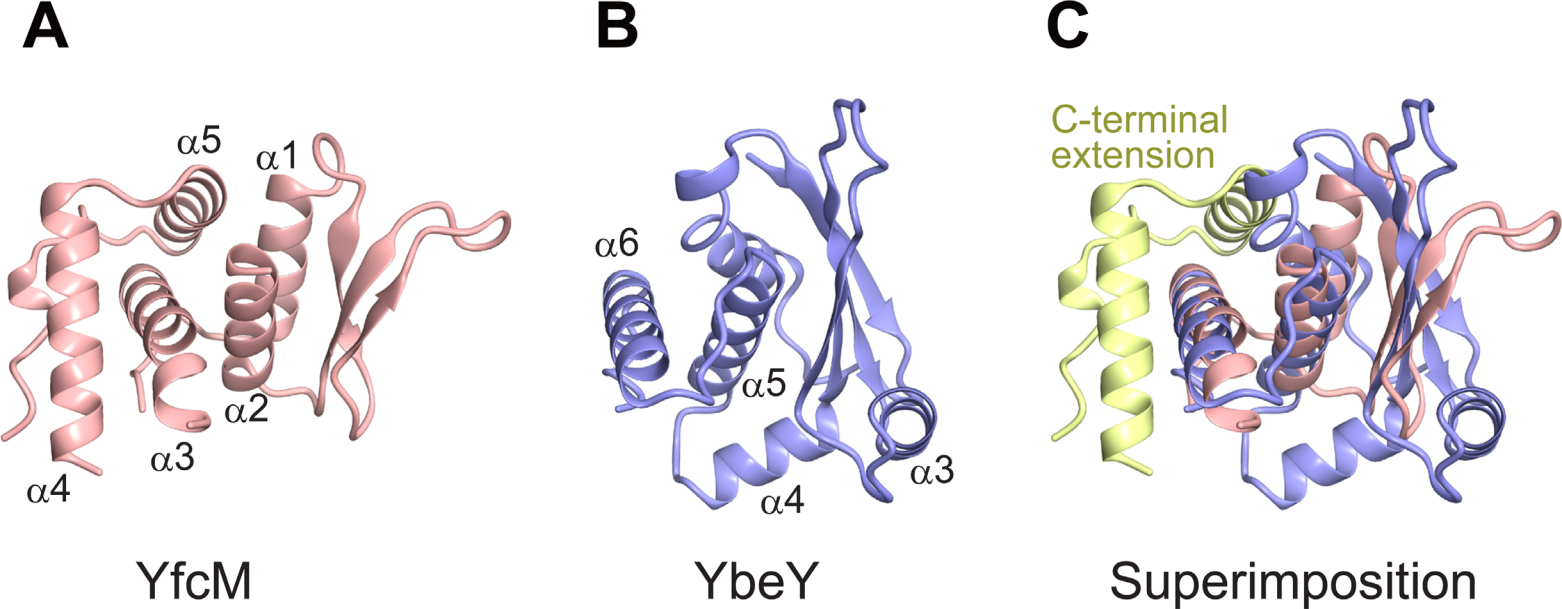
Structural comparison between YfcM and YbeY. (**A**) Structure of YfcM colored pink. (**B**) Structure of YbeY from *E. coli*, colored purple (PDB code: 1XM5). (**C**) Superimposition of the structures of YfcM and YbeY. The C-terminal extension of YfcM is colored yellow.

**Figure 3.**
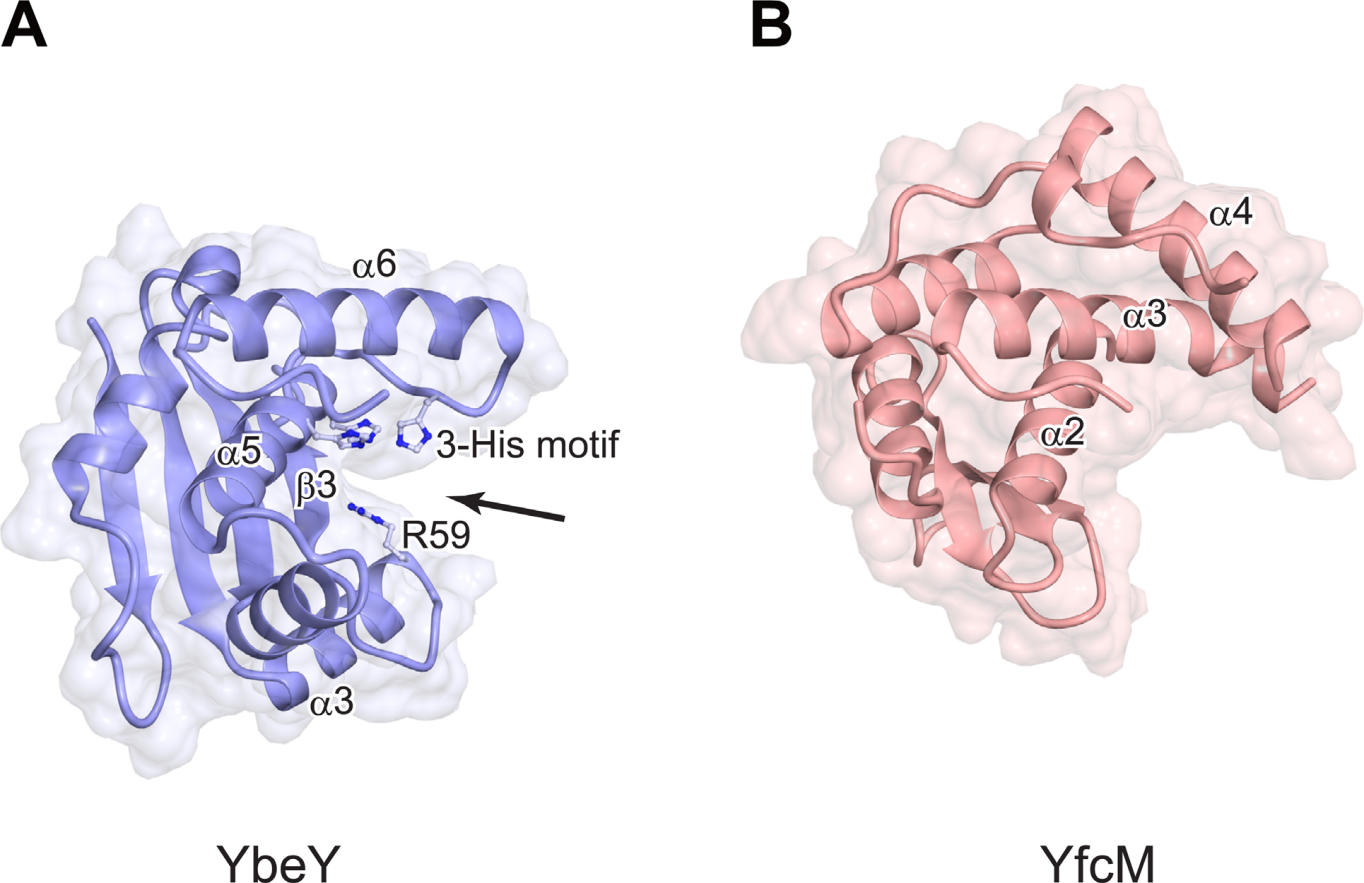
Comparison of the surface models of YbeY and YfcM. (**A**) Ribbon and surface models of YbeY. The catalytic triad formed by three histidine residues (3-His motif) and Arg59 are depicted by ball-and-stick models. The putative catalytic cleft is depicted by an arrow. (**B**) Ribbon and surface models of YfcM.

A superimposition of the structures of YfcM and YbeY revealed that α2 and α3 of YfcM superimpose well on α5 and α6 of YbeY, respectively (Figure 2A, B and C). The root-mean-square (RMS) deviation between the residues from Tyr54 to Gly113, containing α2 and α3 of YfcM, and those from Leu104 to Leu144, containing α5 and α6 of YbeY, is 2.7 Å for all C_α_ atoms. The β1, β2 and β3 strands of YfcM correspond to the β2, β3 and β4 strands of YbeY, respectively, although the strand corresponding to β1 of YbeY is absent in YfcM (Figure 2A, B and C). The α-helix corresponding to α3 of YbeY does not exist in the structure of YfcM (Figure 2A, B and C). In YbeY, α3 forms the putative catalytic cleft, together with β3, α5 and the loop following α5 (41) (Figure 3A). The mutation of the highly conserved Arg59 on α3 generates severe defects in the ribonuclease activity of YbeY (41). The metal-binding three-histidine motif is also located on this cleft (Figure 3A). Due to the absence of the α-helix corresponding to α3 of YbeY, YfcM lacks this cleft (Figure 3B). The absence of the cleft is consistent with the fact that no ribonuclease activity has been reported for YfcM. On the other hand, YfcM harbors the C-terminal extension, consisting of α4, α5 and the following loop, which is absent in YbeY (Figure 2A, B and C). The C-terminal extension contacts α1, α2 and α3 and stabilizes the helices (Supplementary Figure S4A and B). Phe134 and Val138 on α4 hydrophobically interact with Val99 on α3, while Arg137 on α4 forms electrostatic contacts with Asp96 on α3 (Supplementary Figure S4A). Phe157 and Ala160 on α5 form a hydrophobic interaction network with Ile13, Phe14 and Phe18 on α1 and Ile61 and Trp64 on α2 (Supplementary Figure S4B). In addition, the side chain of Tyr165 on α5 forms a hydrogen bond with the side chain of Tyr54 on α2 (Supplementary Figure S4B). The stabilizing effect of the C-terminal extension is consistent with our observation that the deletion of the C-terminal extension of YfcM drastically reduces its solubility (data not shown). Overall, our structural data suggest that YfcM partially adopts the UPF0054 fold and, for the first time, provide evidence that the UPF0054 fold can act as hydroxylase.

### Metal ion-coordinating motif of YfcM

**Figure 4.**
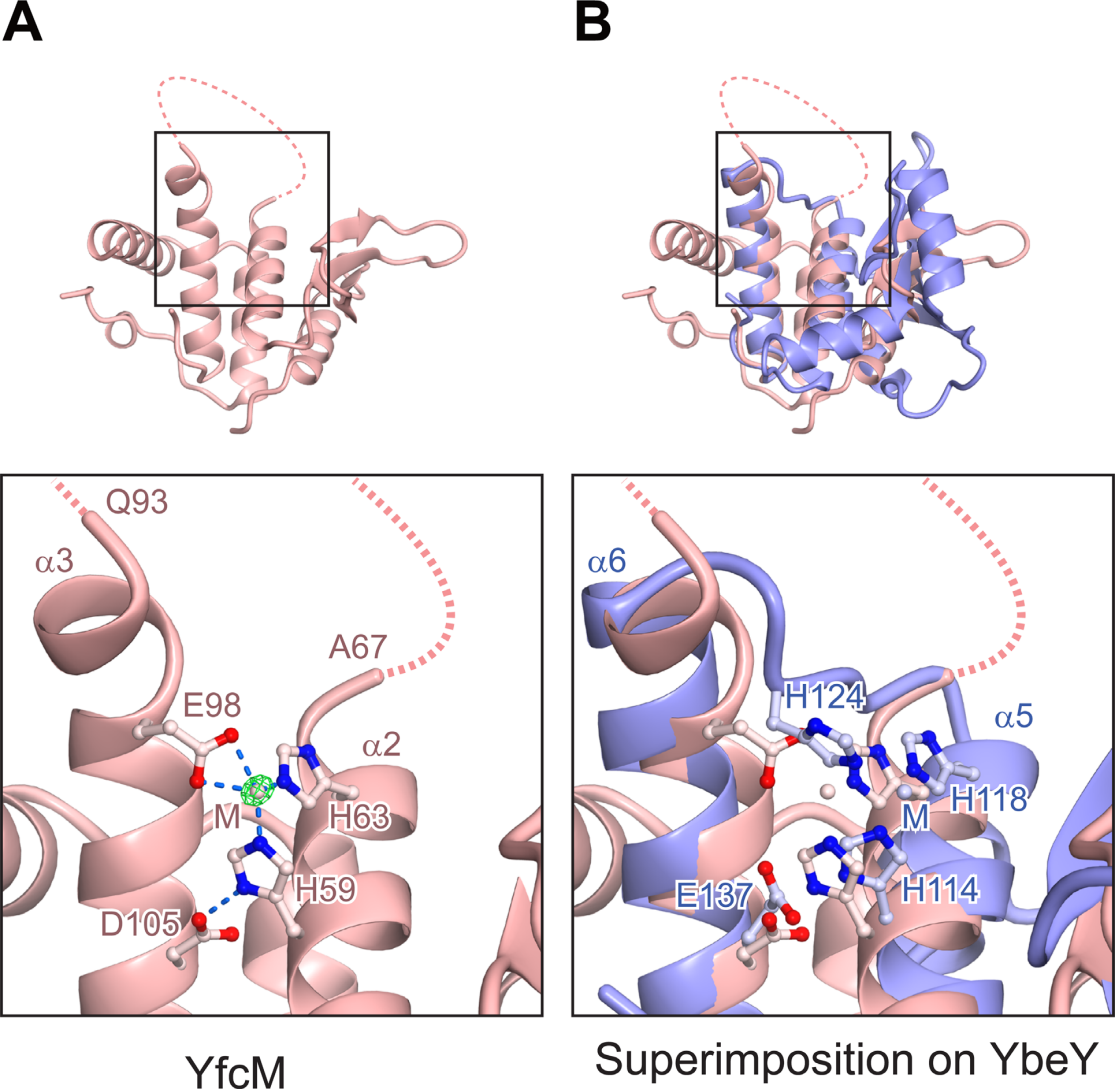
Comparison of the metal-coordination motifs of YbeY and YfcM. (**A**) Metal-coordination motif of YfcM. The F_o_-F_c_ omit map contoured at 10σ is shown in green. Disordered loops are represented by dashed lines. Metal ions are depicted as spheres, labeled M. Electrostatic interactions are represented by dashed blue lines. (**B**) Superimposition of the structures of YfcM and YbeY. The proteins are color-coded as in Figure 2. Residues are depicted by ball-and-stick models.

In the structure of YbeY, the three-histidine motif located on α5 and the loop between α5 and α6 coordinates a metal ion (Figure 3A). The α5 and α6 helices of YbeY structurally correspond to the α2 and α3 helices of YfcM, respectively (Figure 2A, B and C). Our structure of YfcM revealed that it also coordinates a metal ion with His59 and His63 on α2, and Glu98 on α3 (Figure 4A). The metal ion is probably derived from the *E. coli* lysate, but its identity is unclear. Interestingly, the structural superimposition of YbeY and YfcM revealed the nearly identical positions of the metal ion-coordinating residues of these two proteins, with His59, His63 and Glu98 of YfcM corresponding to His114, His118 and His124 of YbeY, respectively (Figure 4B). In a similar manner, Glu137 of YbeY interacts with His114 and helps coordinate a metal ion. Likewise, Glu137 corresponds to Asp105 of YfcM, which interacts with His59 (Figure 4B). The interaction between Asp 105 and His59 may help coordinate Glu98 to the metal ion by bringing α3 into closer proximity to α2. A sequence alignment of the metal binding motif revealed that His59, His63 and Glul98 are all highly conserved in YfcM (Supplementary Figure S2). Asp105 is also conserved as the acidic residue (aspartate or glutamate) (Supplementary Figure S2). Therefore, YfcM harbors a metal ion-coordinating motif consisting of two histidines and a glutamate, and their positions overlap with those of the metal ion-coordinating motif in YbeY.

### YfcM encodes an active canonical 2-His-1-carboxylate motif

The metal ion-coordinating motif in YfcM is classified as a 2-His-1-carboxylate motif. The 2-His-1-carboxylate motif consists of two histidine residues and one aspartate or glutamate residue, and coordinates an Fe(II) ion (27). The 2-His-1-carboxylate motif is highly conserved among non-heme iron enzymes, many of which catalyze oxidation or oxidation-type reactions, such as hydroxylation, cyclization, ring expansion, epoxidation, etc. (27,42). In these oxidative reactions, the Fe(II) ion coordinated by the 2-His-1-carboxylate motif binds and activates the substrate along with molecular oxygen, which ultimately enables these reactions (27,42). Therefore, it is possible that His59, His63 and Glu98 of YfcM form a 2-His-1-carboxylate motif for the coordination of an Fe(II) ion, which binds to EF-P and molecular oxygen for the hydroxylation of Lys34 of EF-P, as in the cases of other non-heme iron enzymes.

**Figure 5.**
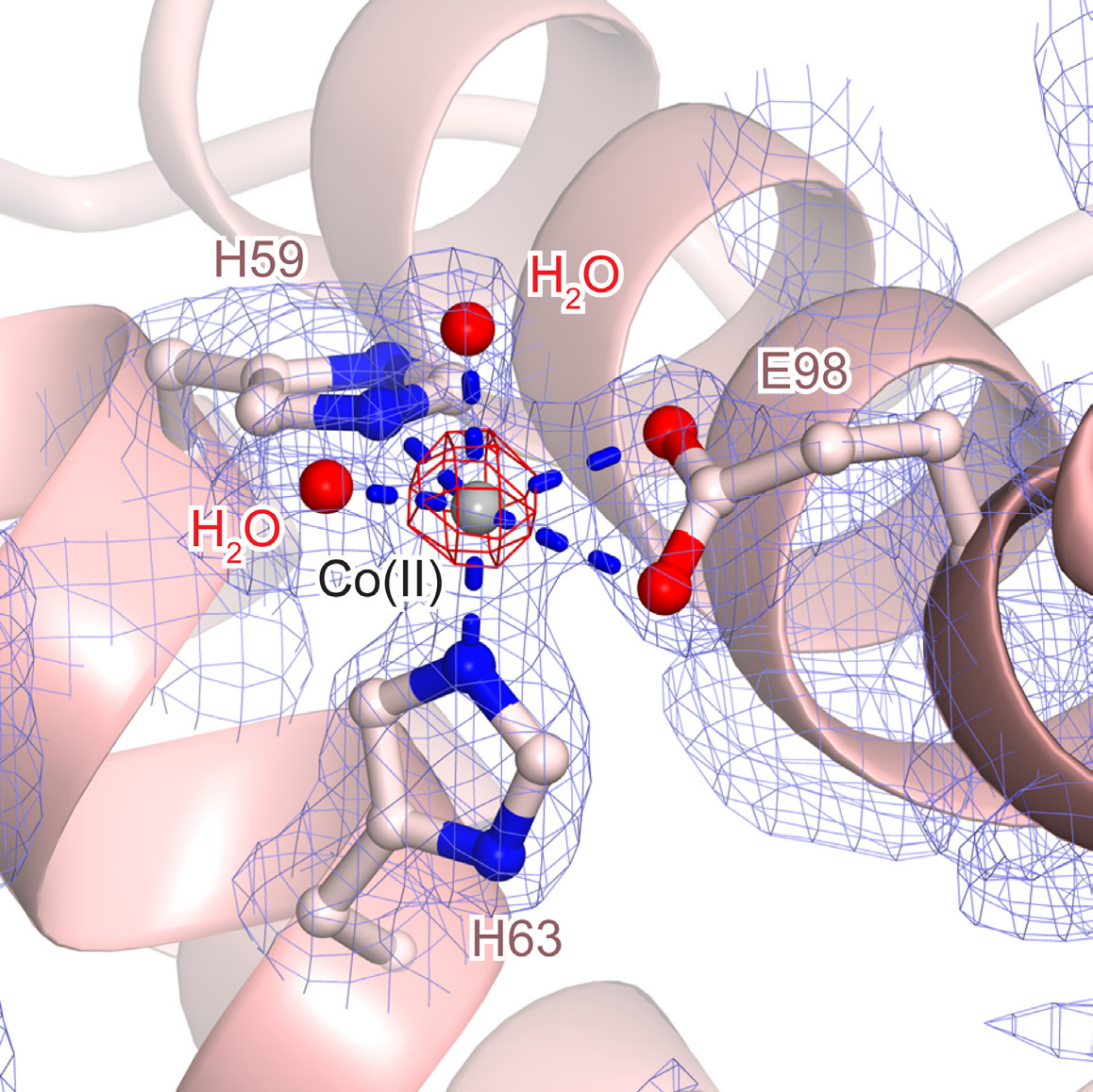
Co(II) ion coordination by YfcM. The 2F_o_-F_c_ electron density map contoured at 1.0σ and the anomalous difference Fourier map contoured at 20σ are shown in blue and red, respectively. The bound Co(II) ion and water molecules are depicted by gray and red spheres, respectively. Residues involved in the coordination of the Co(II) ion are depicted by ball-and-stick models.

We tested whether an Fe ion binds to YfcM. The purified YfcM protein was mixed with FeSO_4_, and the free FeSO_4_ was removed by gel-filtration. The fraction containing YfcM was analyzed by atomic absorption spectrometry. The results showed that an Fe ion coeluted with YfcM from the gel-filtration column, and the molar ratio of YfcM and Fe was 1 : 0.37 (Fe-bound, Supplementary Table S3). Therefore, the Fe ion directly binds to YfcM. In contrast, an Fe ion was not observed in the purified YfcM in the absence of exogenous Fe ion (Apo, Supplementary Table S3). This may be because the Fe(II) ion bound to YfcM was oxidized to an Fe(III) ion during the purification process. The ionic radius change would destabilize the interaction between the Fe(III) ion and YfcM, leading to the dissociation of the Fe(III) ion from YfcM. The metal ion observed in the structure of YfcM could be another metal ion, such as Mg(II), which may have bound during the purification process. In order to confirm that an Fe(II) ion is coordinated by the putative 2-His-1-carboxylate motif of YfcM, we prepared crystals of Co(II)-bound YfcM and calculated the anomalous difference Fourier map, using the data set collected at the peak wavelength of the Co K-shell absorption edge (1.6049 Å). The Co(II) ion has an ionic radius similar to that of an Fe(II) ion (43) and can adopt the geometry of an octahedral complex together with six ligands, similar to an Fe(II) ion (43). Therefore, a Co(II) ion often binds to the 2-His-1-carboxylate motif in a manner that mimics an Fe(II) ion. In fact, it was reported that the substitution of the Fe(II) ion for the Co(II) ion in the 2-His-1-carboxylate motif of homoprotocatechuate 2,3-dioxygenase from *Brevibacterium fuscum* (HPCD) has almost no effect on the arrangement of metal ion-coordinating residues and the overall structure of HPCD (44). Furthermore, a Co(II) ion occupies an Fe(II) ion-coordinating site more stably than an Fe(II) ion, because an Fe(II) ion is easily air-oxidized to an Fe(III) ion, thus changing its ionic radius. The resultant electron density map clearly exhibited a strong Co(II) peak at the metal-coordinating site, indicating that a Co(II) ion can be coordinated by His59, His63 and Glu98 (Figure 5). In this structure, six atoms participate in the Co(II) ion coordination (nitrogen atoms (NEs) of His59 and His63, two oxygen atoms (OEs) of Glu98 and two oxygen atoms of two water molecules; distances from the Co(II) ion are summarized in Supplementary Table S5), which may mimic the Fe(II) ion coordination (Figure 5). This observation further indicated that His59, His63 and Glu98 can coordinate an Fe(II) ion. Therefore, our data suggested that His59, His63 and Glu98 of YfcM form the putative 2-His-1-carboxylate motif for the coordination of an Fe(II) ion, as in the active sites of non-heme iron enzymes.

### The hydroxylation activity of YfcM is mediated by metal ion catalysis

**Figure 6.**
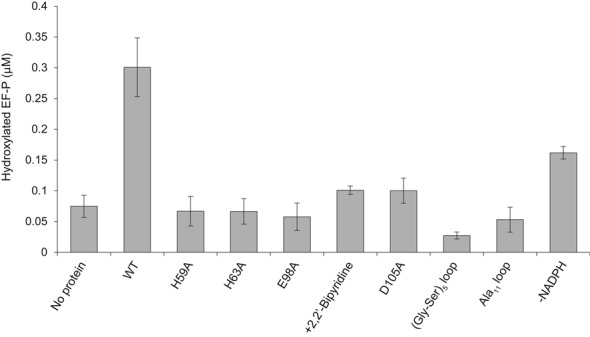
Hydroxylation in Lys34 of β-lysylated EF-P by WT and variants of YfcM. Error bars represent the standard deviations from three independent experiments.

The putative 2-His-1-carboxylate motif of YfcM is considered to form the active site of YfcM (Figure 5). In order to determine the importance of this motif, we performed *in vitro* hydroxylation assays for Lys34 of β-lysylated EF-P by YfcM. First, we carried out the assay based on the previous study (19) using the WT YfcM protein. The reaction mixture contained 5 μM YfcM, 5 μM β-lysylated EF-P, an essentially protein-free filtrate from the *E. coli Δyfcm* strain, 10 μM FeSO_4_, 5 mM NADPH and other redox active metabolites, unless otherwise stated (see above). The reaction mixture was incubated for 155 min at 30°C, and the hydroxylation efficiency was estimated from the mass spectrometry results. This hydroxylation efficiency of WT YfcM was 7.95% (Supplementary Figure S3), which is much lower than that reported previously (∼60%) (19). This may be because of the lower concentration of YfcM and β-lysylated EF-P in our experiment (10 μM YfcM and 15 μM β-lysylated EF-P were used in the previous study (19)) and subtle differences in the experimental conditions (for instance, the amount of protein-free filtrate). Next, we prepared mutant YfcM proteins, in which the residues forming the putative 2-His-1-carboxylate motif were replaced with alanine (H59A, H63A and E98A), and performed the hydroxylation assays. We confirmed that all of the YfcM proteins used for our *in vitro* hydroxylation assays were highly purified and were not aggregated, by sodium dodecyl sulphate-polyacrylamide gel electrophoresis and gel-filtration analyses (Supplementary Figure S5A and B). Our results indicated that the hydroxylation efficiencies of these mutant YfcM proteins were drastically reduced, in comparison to WT YfcM (Figure 6 and Supplementary Table S7). The hydroxylation efficiencies of these YfcM mutants were as low as the background level (No protein, Figure 6 and Supplementary Table S7). Although some hydroxylated EF-P was detected in the absence of protein (No protein, Figure 6 and Supplementary Table S7), this may be because some of the expressed EF-P was modified by endogenous PoxA and YfcM in *E. coli* and contaminated the purified EF-P. Moreover, the hydroxylation efficiency of WT YfcM was reduced by adding 200 μM of the iron chelator 2,2′-Bipyridine, instead of 10 μM FeSO_4_ (+2,2′-Bipyridine, Figure 6 and Supplementary Table S7, see also Materials and Methods), possibly because 2,2′-Bipyridine removed the Fe(II) ion of YfcM. The mutation of YfcM Asp105 to Ala also caused a 3-fold reduction in its hydroxylation activity (D105A, Figure 6 and Supplementary Table S7). Since Asp105 interacts with His59 and may facilitate the coordination of Glu98 to an Fe(II) ion (Figure 4A), the D105A mutation may destabilize the conformation of the 2-His-1-carboxylate motif. These results support our hypothesis that the putative 2-His-1-carboxylate motif of YfcM plays an essential role in the hydroxylation of Lys34 of β-lysylated EF-P. Therefore, as in the cases of other non-heme iron enzymes, the putative 2-His-1-carboxylate motif is considered to form the active site of YfcM. Interestingly, the predicted model of human DOHH, which hydroxylates the 4-amino butyl moiety attached to eIF5A, may also harbor the 2-His-1-carboxylate motif (25). In addition, the mutation of the glutamate residue predicted to coordinate an Fe(II) ion in *Schizosaccharomyces pombe* DOHH generates a temperature-sensitive phenotype with disrupted morphology and aberrant distribution of mitochondria at nonpermissive temperatures (25,45). These observations suggested that the highly conserved 2-His-1-carboxylate motif plays an important role in the function of the eukaryotic DOHH.

The role of the disordered loop between α2 and α3 of YfcM (residues Ala67 to Gln93), which is located near the putative 2-His-1-carboxylate motif, was also investigated (Figure 4A). The amino acid sequence of this loop is highly conserved (Supplementary Figure S2). We prepared mutant YfcM proteins, in which the residues from Gly68 to Ser92 were replaced with either a 5 repeat linker sequence ((Gly-Ser)_5_ loop) or 11 alanine residues (Ala_11_ loop), and measured the hydroxylation efficiencies of these variants. These mutations also produced severe defects in the hydroxylation efficiency of YfcM, suggesting the important role of this loop ((Gly-Ser)_5_ loop and Ala_11_ loop, Figure 6 and Supplementary Table S7). Given that this loop is much longer than the corresponding loop of YbeY (27 residues in YfcM; 7 residues in YbeY) (Figure 4A and B), one possible functional role of this loop could be the recognition of β-lysylated EF-P.

Furthermore, previous studies reported that the Lys34 hydroxylation of β-lysylated EF-P by YfcM is stimulated in the presence of NADPH (19). In the absence of 5 mM NADPH, we observed a 2-fold decrease in the hydroxylation activity (-NADPH, Figure 6 and Supplementary Table S7), suggesting that this redox active reagent is also required for the reaction at the 2-His-1-carboxylate motif of YfcM.

## DISCUSSION

The structural features of YfcM are distinct from those of other amino acid hydroxylases. We compared the YfcM structure with previously reported amino acid hydroxylase structures. The catalytic domains of the aromatic amino acid hydroxylases, including the phenylalanine, tyrosine and tryptophan hydroxylases (PheOH, TyrOH and TrpOH, respectively), are highly conserved. They consist mainly of helices and loops in a basket-like arrangement (46–48). Other hydroxylases adopt the β jellyroll fold, consisting of double β-strands (49–51). However, the structure of YfcM is entirely different from these previously reported amino acid hydroxylase structures, suggesting that alternative three-dimensional folds exist for hydroxylases. Consistent with the structural difference, YfcM may hydroxylate Lys34 of EF-P in a different manner. The aromatic amino acid hydroxylases hydroxylate amino acids using tetrahydrobiopterin as a cofactor (46–48), while the β jellyroll fold hydroxylases do so in a 2-oxoglutarate-dependent manner (49–51). In contrast, YfcM may hydroxylate Lys34 of β-lysylated EF-P using NADPH as a cofactor, as the hydroxylation was stimulated in the presence of NADPH (19) (Figure 6 and Supplementary Table S7). The structure of YfcM also differs from the previously reported model of human DOHH (25). DOHH hydroxylates the 4-amino butyl moiety attached to eIF5A, the eukaryotic homolog of EF-P (25). DOHH is considered to be a HEAT repeat-containing protein, harboring two bundles of four HEAT repeats linked by a loop (25). This indicated that the hydroxylation of EF-P by YfcM also occurs in a different manner from that of eIF5A by DOHH, although EF-P and eIF5A are related (8,9,12,52–54).

Instead, YfcM structurally resembles UPF0054 family proteins. The general functions of the UPF0054 family proteins remain unknown. Although the structures of the UPF0054 family proteins are similar to those of the matrix metalloproteinases with the conserved three-histidine motif coordinating a metal ion (37), no proteinase activity has been detected in the UPF0054 family proteins to date. Previous studies revealed several functions of a member of the UPF0054 family, YbeY. YbeY is a highly conserved heat shock protein involved in transcription and translation (55–58). YbeY is also thought to be involved in the bacterial small RNA pathway, based on the observation that the sequence and structure of YbeY are similar to those of the Argonaute MID domain (40,59). Furthermore, YbeY has a single strand-specific endoribonuclease activity (41,60,61). Together, YbeY and RNase R perform the maturation of 16S ribosomal RNA and the degradation of 70S ribosomes containing a defective 30S subunit (41,60). The ribonuclease activity of YbeY is reduced in the presence of ethylenediaminetetraacetic acid or when the histidine residues involved in metal ion coordination are mutated to alanine, indicating that YbeY is a metal-dependent ribonuclease (41). Therefore, the UPF0054 family proteins are considered to be metalloproteins involved in RNA metabolism. The structural similarity of YfcM to UPF0054 family proteins indicated that the UPF0054 fold may also act as a hydroxylase (Figure 2A, B and C). Furthermore, the metal ion-coordinating three-histidine motif of YbeY is replaced with the putative 2-His-1-carboxylate motif in YfcM, which is an Fe(II)-coordinating motif characteristic of non-heme iron enzymes (Figure 4A and B). Our atomic absorption spectrometry and X-ray crystallographic analyses confirmed the interaction of an Fe ion with YfcM, and the binding of a Co(II) ion to the putative 2-His-1-carboxylate motif of YfcM in a manner mimicking an Fe(II) ion (Figure 5, Supplementary Tables S3 and S5). These observations indicate that the hydroxylase activity of YfcM is accomplished by replacing the metal ion-coordinating three-histidine motif of YbeY with a 2-His-1-carboxylate motif. This proposal is supported by the results from our *in vitro* hydroxylation assay, showing that mutations in the putative 2-His-1-carboxylate motif of YfcM drastically reduce the hydroxylation efficiency of YfcM (Figure 6 and Supplementary Table S7). Furthermore, the addition of the iron chelator 2,2′-Bipyridine and the absence of FeSO_4_ also repressed the hydroxylation efficiency of YfcM, possibly by removing the Fe(II) ion from the putative 2-His-1-carboxylate motif of YfcM (Figure 6 and Supplementary Table S7).

We then considered the hydroxylation mechanism of Lys34 of EF-P by YfcM. Given that the hydroxylation efficiency of WT YfcM is enhanced in the presence of NADPH (WT and -NADPH, Figure 6 and Supplementary Table S7) (19), we hypothesized that NADPH is involved in the transfer of electrons to the 2-His-1-carboxylate motif of YfcM. In an analogous manner, the active site of the naphthalene 1,2-dioxygenase (NDO) from *Pseudomonas sp.* receives electrons from NAD(P)H via ferredoxin_NAP_ reductase and ferredoxin_NAP_, and converts naphthalene to (+)-*cis*-(1*R*,2*S*)-dihydroxy-1,2-dihydronaphthalene, using an Fe(II) ion coordinated by a 2-His-1-carboxylate motif (62–67) (Supplementary Figure S6A and B). One of the histidine residues in the 2-His-1-carboxylate motif (His208) interacts with Asp205 (64) (Supplementary Figure S6B). Asp205 also interacts with His104 of the neighboring subunit, which coordinates the [2Fe-2S] cluster (64) (Supplementary Figure S6B). This [2Fe-2S] cluster receives electrons from ferredoxin_NAP_ for the hydroxylation of naphthalene (62–67). Therefore, the electrons received from ferredoxin_NAP_ are considered to be transferred from the [2Fe-2S] cluster to the Fe(II) ion of the neighboring subunit via Asp205 (64). Based on this observation, it is possible that the electrons received from NADPH could be transferred to the active site of YfcM via Asp105 (Figure 4A). The interaction of Asp105 with His59 could not only stabilize the conformation of the 2-His-1-carboxylate motif of YfcM but also form the pathway for electron transfer. However, the similarity of the active site structures between YfcM and NDO raises the possibility that YfcM receives electrons from NADPH through other components. In fact, our *in vitro* binding assays could not detect a direct interaction between YfcM and NADPH (data not shown), making it unlikely that YfcM directly uses NADPH as the cofactor for the hydroxylation reaction, although it enhances the reaction. Furthermore, the hydroxylation of Lys34 of β-lysylated EF-P has been reported only in the presence of an essentially protein-free filtrate of the *E. coli Δyfcm* strain (19), suggesting that components in the filtrate could also be required for the hydroxylation reaction. Therefore, it is likely that the hydroxylation of Lys34 in β-lysylated EF-P is catalyzed by YfcM and other unknown components *in vivo*. In addition, the hydroxylation activity of YfcM in our assay seems to be much lower than that of other hydroxylases. For instance, 2.8 μM TYW5, a β jellyroll fold hydroxylase, hydroxylates more than 90% of its substrate (more than 540 nM of 600 nM tRNA^Phe^ with 7-(α-amino-α-carboxypropyl)wyosine) in 1 h (68). In contrast, in our assay of YfcM, 5 μM YfcM hydroxylated less than 8% of 5 μM β-lysylated EF-P (less than 400 nM of β-lysylated EF-P) in 155 min (Supplementary Figure S3 and Table S7). Although we cannot directly compare these results because of the different experimental conditions, our findings suggest that YfcM has lower hydroxylation activity as compared with other hydroxylases under our assay conditions, implying the requirement for other components for the efficient hydroxylation of β-lysylated EF-P by YfcM.

## CONCLUSION

We have presented the crystal structure of *E. coli* YfcM. Whereas the overall structure of YfcM is different from those of other known hydroxylases, it is similar to those of the UPF0054 family proteins, especially the ribonuclease YbeY. Although the catalytic cleft of YbeY is not conserved in YfcM, YfcM shares a similar metal ion-coordinating motif with YbeY. This metal ion-coordinating motif of YfcM resembles the canonical 2-His-1-carboxylate motif for the coordination of an Fe(II) ion, which is highly conserved in the non-heme iron enzymes. Our *in vitro* hydroxylation assay confirmed that the putative 2-His-1-carboxylate motif of YfcM plays an essential role in the hydroxylation of Lys34 in β-lysylated EF-P. However, the detailed reaction mechanism, including how YfcM recognizes the Lys34 of β-lysylated EF-P and what other components are necessary to hydroxylate EF-P, remains to be determined. Finally, the elucidation of other cofactors required for the hydroxylation of EF-P may provide a link to the functional role of the hydroxyl moiety of Lys34 in EF-P.

## ACCESSION NUMBERS

The atomic coordinates and structure factors have been deposited in the Protein Data Bank, www.pdb.org (PDB codes: 4PDN for native form and 3WTR for Co(II)-bound form).

## SUPPLEMENTARY DATA

Supplementary Data are available at NAR Online.

SUPPLEMENTARY DATA
