# Supplementary material for: The non-canonical hydroxylase structure of YfcM reveals a metal ion-coordination motif required for EF-P hydroxylation
Source: Nucleic Acids Res. 2014 Oct 1;42(19):12295–305. doi: 10.1093/nar/gku898 (PMC4231759; doi:10.1093/nar/gku898)
Supplement: SUPPLEMENTARY DATA [file supp_42_19_12295__index.html]

The non-canonical hydroxylase structure of YfcM reveals a metal ion-coordination motif required for EF-P hydroxylation — The non-canonical hydroxylase structure of YfcM reveals a metal ion-coordination motif required for EF-P hydroxylation — SUPPLEMENTARY DATA 

# The non-canonical hydroxylase structure of YfcM reveals a metal ion-coordination motif required for EF-P hydroxylation

## SUPPLEMENTARY DATA

**Files in this Data Supplement:**

- SUPPLEMENTARY DATA
