# Supplementary material for: The non-canonical hydroxylase structure of YfcM reveals a metal ion-coordination motif required for EF-P hydroxylation
Source: Nucleic Acids Res. 2014 Oct 1;42(19):12295–305. doi: 10.1093/nar/gku898 (PMC4231759; doi:10.1093/nar/gku898)
Supplement: SUPPLEMENTARY DATA [file supp_gku898_nar-01062-r-2014-File008.pdf]

# Supplementary data

## Supplementary methods

### Identification and estimation of EF-P hydroxylation by LC-MS/MS

Approximately 500 ng of peptides were separated by reverse-phase HPLC (Dionex, Waltham, MA) on a 0.2 mm×150 mm C18AQ column (3 μm, 200 Å, Michrom Bioresources Inc., Auburn,CA) coupled to either an LTQ Orbitrap XL mass spectrometer (ThermoFisher Scientific, Waltham, MA) for protein and peptide identification or an LTQ linear ion trap (Thermo Scientific) for estimation of lysine hydroxylation. In all cases, peptide separation was accomplished with water (A) and acetonitrile (B) with the addition of 0.1% formic acid as an ion-pairing agent. Peptides were first loaded onto a 0.3 mm×5 mm Acclaim PepMap100 C18 trap cartridge (5 μm, 100 Å, Dionex, Waltham, MA) and washed with 5% B for 3 minutes. Peptides were eluted at a flow rate of 2 μl/min with an increasing linear gradient of B from 5% to 30% over 47 minutes. The column was subsequently washed with 90% B for 5 minutes and the system was equilibrated for 10 minutes prior to the following injection.

An LTQ Orbitrap XL mass spectrometer was used to identify both the modified and unmodified forms of the *Escherichia coli* EF-P protein. Peptides were ionized using a captive spray ionization source (Michrome Bioresources Inc., Auburn, CA) with an ionization voltage and capillary temperature of 2.0 kV and 175°C, respectively. Positive ion data acquisition was performed in a data-dependent fashion, with the dynamic exclusion and preview modes enabled. The top-5 precursor ions were selected for fragmentation with dynamic exclusion settings as follows: repeat count = 2, repeat duration = 20 s, exclusion list size = 100, exclusion duration = 60 s and exclusion mass width of ±1.50 m/z. Precursor ions underwent CID fragmentation in the LTQ linear ion trap with a normalized collision energy (NCE) of 35%. The raw data were converted to mzXML files using MSConvert and searched with MassMatrix (1,2) against a UniProt *E. coli* K12 proteome concatenated with modified forms of the EF-P sequence. This search confidently identified the (*R*)-β-lysyl-lysine and (*R*)-β-lysyl-hydroxylysine forms of EF-P. Once the protein identities were obtained, the experiment was repeated using the precursor ion inclusion mass list containing the precursor masses of the target peptides in multiple charged states. The instrument was operated in the orbitrap-orbitrap mode, in which both the precursor and product ions were detected in the orbitrap MS. This method was used to validate the identity of the obtained high mass resolution and the accuracy of the precursor and fragment ions for the (*R*)-β-lysyl-lysine and (*R*)-β-lysyl-hydroxylysine EF-P. The precursor and fragment ion resolutions were set at 15,000 and 7,500, respectively. Peptides bearing either (*R*)-β-lysyl-lysine or (*R*)-β-lysyl-hydroxylysine at position 34 and either a normal or oxidized Met16 were identified and manually validated (Table S6). The combination of chromatographic separation and unique fragment ions allowed for highly

specific and selective identification of the four modified forms of EF-P.

Once highly confident precursor – product ion transitions were determined, follow-up parallel reaction monitoring (PRM) experiments were performed on an LTQ linear ion trap (Thermo Scientific, Waltham, MA) (3). This experiment leverages the faster scan rate of the ion trap and allows for the estimation of the conversion of (*R*)-β-lysyl-lysine to (*R*)-β-lysyl-hydroxylysine EF-P. Again, the peptides were ionized using a captive spray ionization source (Michrome Bioresources Inc., Auburn, CA) with an ionization voltage and capillary temperature of 2.0 kV and 200°C, respectively. To perform the PRM experiments, a target mass inclusion list containing the multiple charge states of the precursor ions was used, as described above. The precursor ions were selected with an isolation width of 1.5 Da and fragmented via CID (NCE 35%), and all fragment ion masses were collected. To differentiate between lysine hydroxylation and methionine oxidation, extracted ion chromatograms (XIC) were produced from the +3 species containing unique product ion transitions (Figure S3 and Table S6). Finally, the yield of (*R*)-β-lysyl-hydroxylysine EF-P was estimated by the relative comparison of the corresponding XIC peaks. Quantification was performed using Thermo Xcalibur version 2.0, with Genesis algorithm peak detection and smoothing of 5.

## Supplementary figures

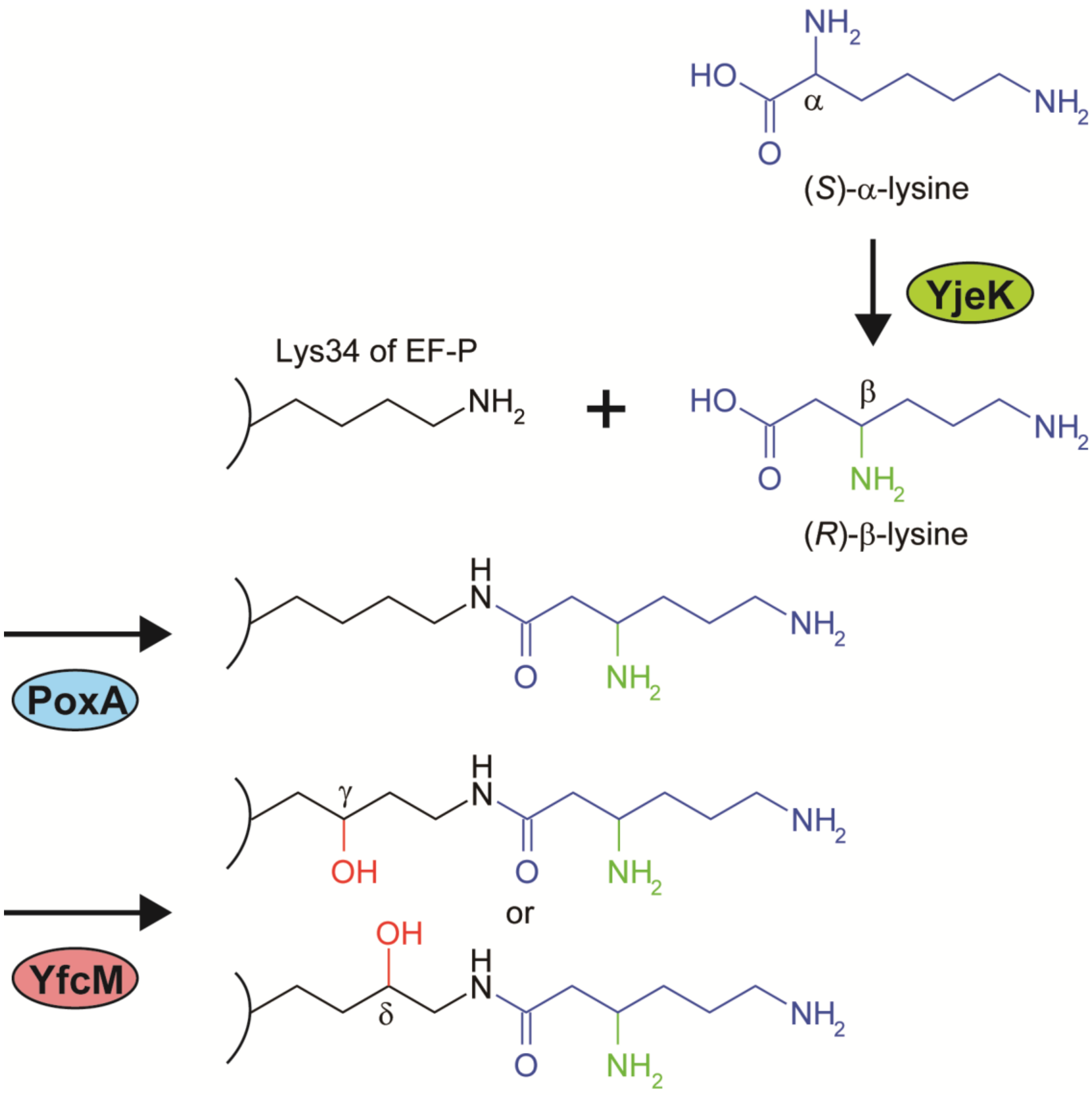


**Figure S1 Post-translational modification pathway of Lys34 of EF-P.**

The overview of the post-translational modification pathway of Lys34 of EF-P is shown (4-6). First, the (*S*)-α-lysine is converted to (*R*)-β-lysine by YjeK. Second, the (*R*)-β-lysine is attached to the ε-amino group of Lys34 of EF-P by PoxA, a paralogous protein of lysyl-tRNA synthetase. Finally, Lys34 of EF-P is hydroxylated by YfcM at its C4(γ) or C5(δ).

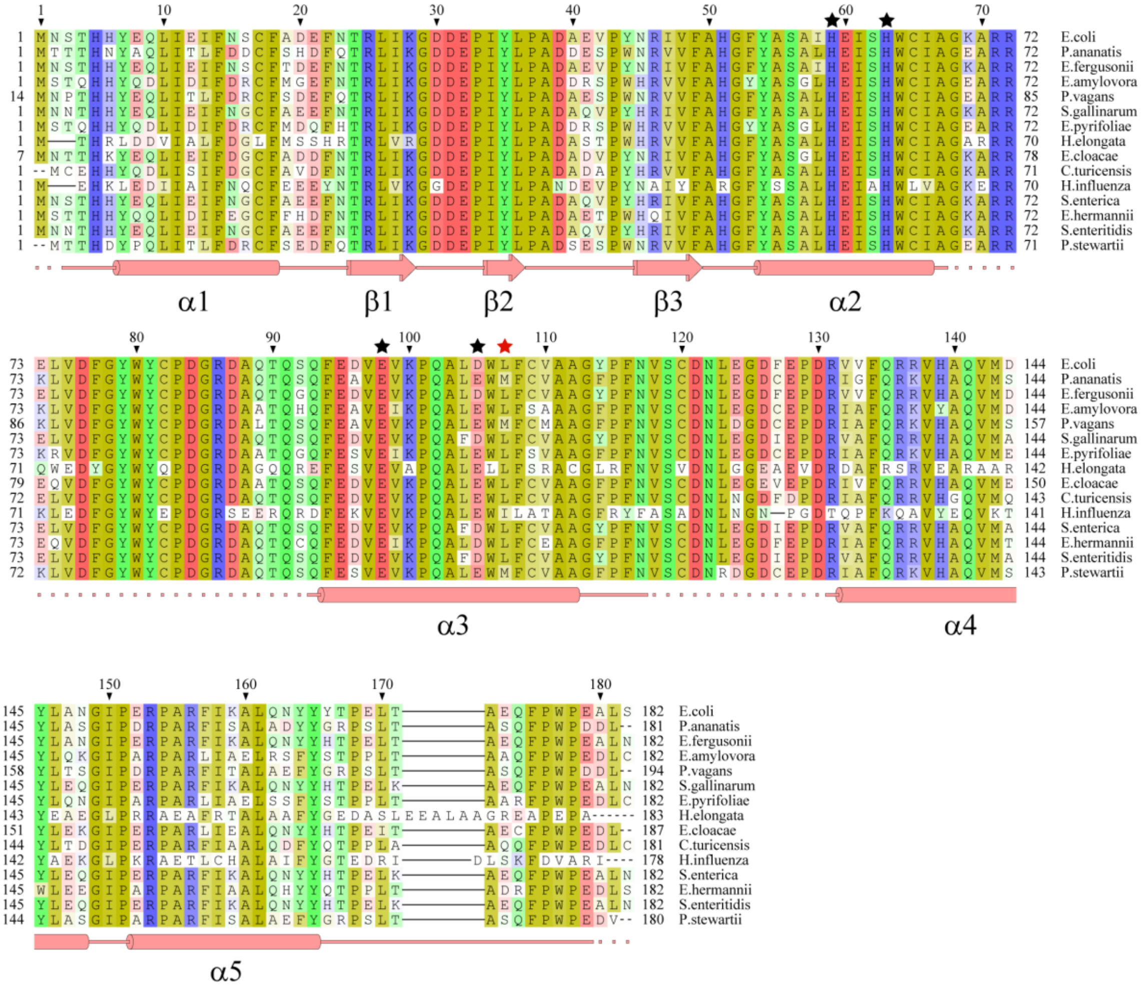

**Figure S2 Amino acid sequence alignment of YfcM proteins.**

The numbering is based on the *E. coli* YfcM protein. The secondary structures and their names in the *E. coli* YfcM protein are shown below the sequences. Conserved basic residues are highlighted in blue, acidic in red, hydrophilic in green, and hydrophobic in dark yellow. Residues discussed in the main text are marked with black stars. Leu107, which was mutated to Met in SeMet-labeled YfcM is marked with a red star. The sequence alignment was generated with the program PROMALS3D (7).

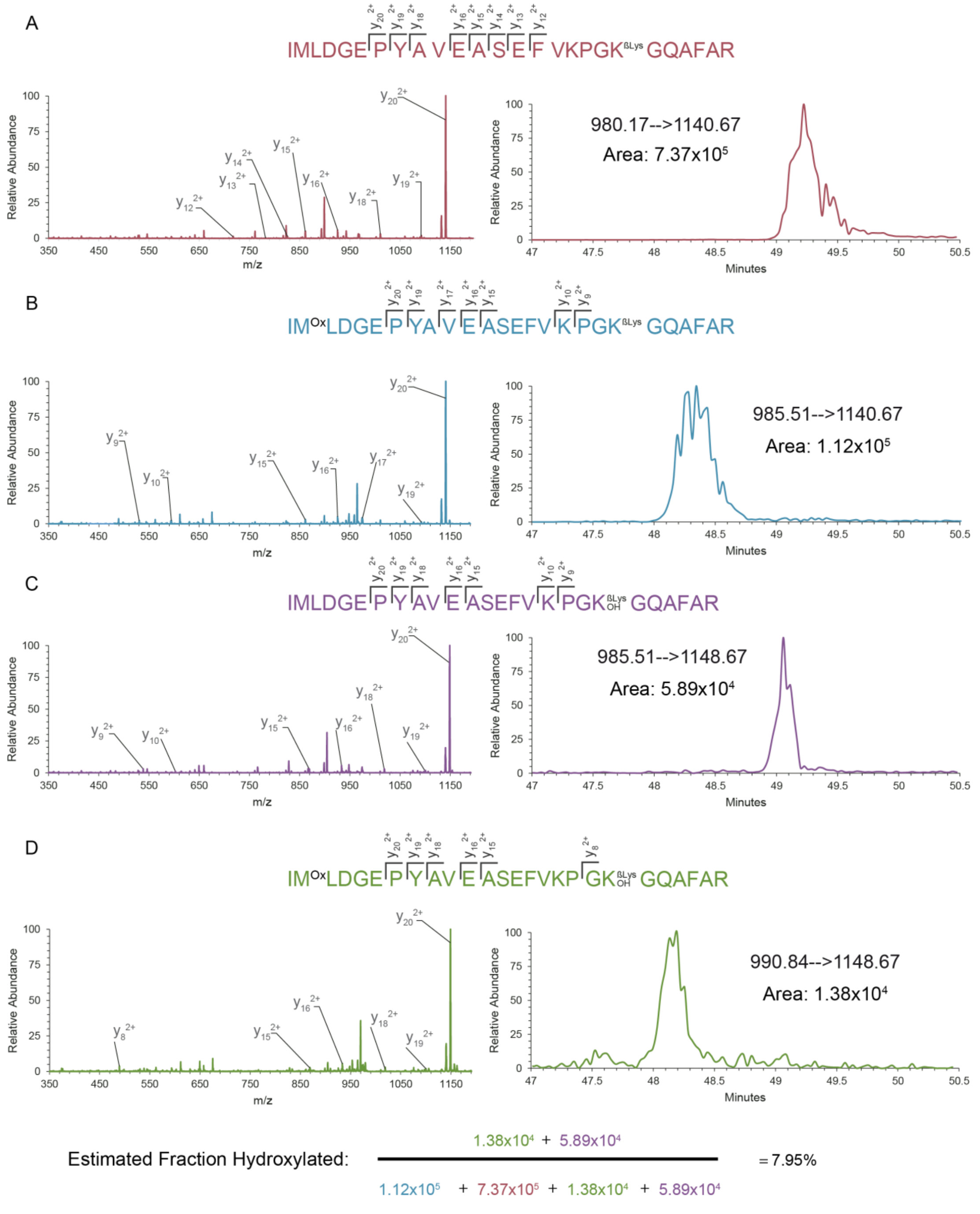


**Figure S3 Quantification of EF-P hydroxylation.**

Parallel reaction monitoring fragmentation spectra (left panel) and extracted ion chromatograms (right panel) for the hydroxylation of (*R*)-β-Lysyl-EF-P by WT YfcM. (A) Non-hydroxylated peptide, (B) non-hydroxylated, methionine oxidized peptide, (C) hydroxylated peptide and (D) hydroxylated and methionine oxidized peptide. Areas under the curves were quantified for each transition to calculate the percentage of hydroxylated peptide (bottom section).

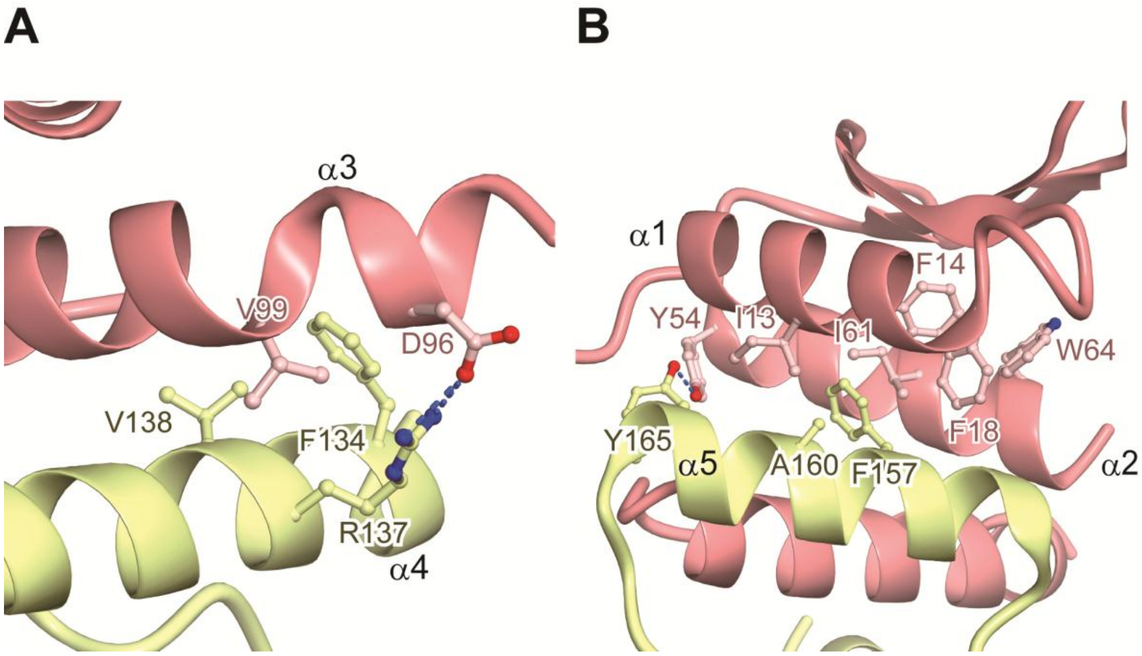


**Figure S4 C-terminal extension involved in intramolecular contacts in YfcM.**

(A) Interaction of α4 with α3.

(B) Hydrophobic interaction network formed by α1, α2 and α5.

The YfcM protein is color-coded as in Figure 2C.

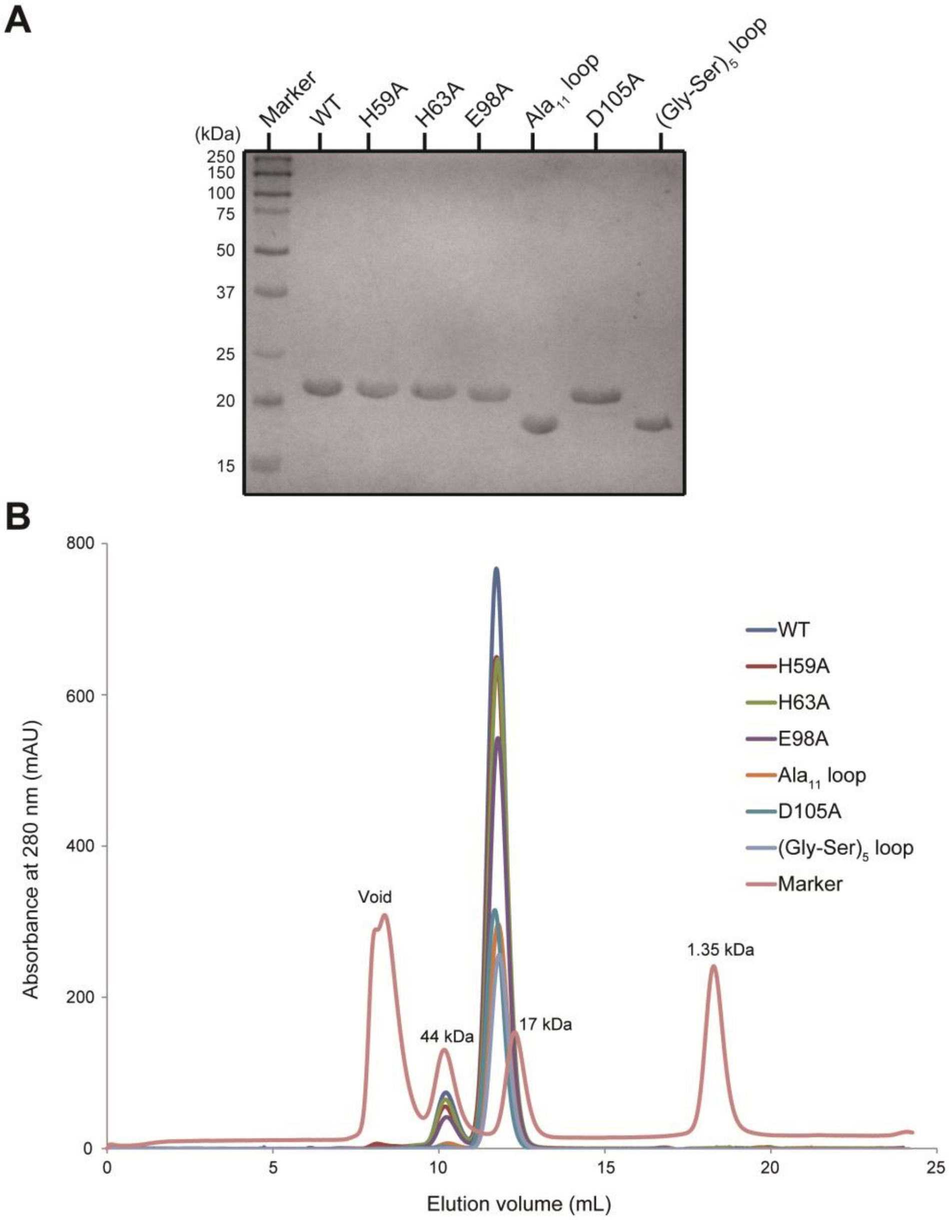


**Figure S5 YfcM proteins prepared for the *in vitro* hydroxylation analysis.**

Analyses by 13% SDS-PAGE (A) and gel-filtration (B) of YfcM proteins prepared for the *in vitro* hydroxylation analysis. A Superdex 75 10/300 column (GE Healthcare) was used for the gel filtration. The Precision Plus Protein™ Kaleidoscope™ standards (BIO-RAD) and Gel Filtration Standard (BIO-RAD) were used as the molecular weight markers for SDS-PAGE and gel-filtration analyses, respectively.

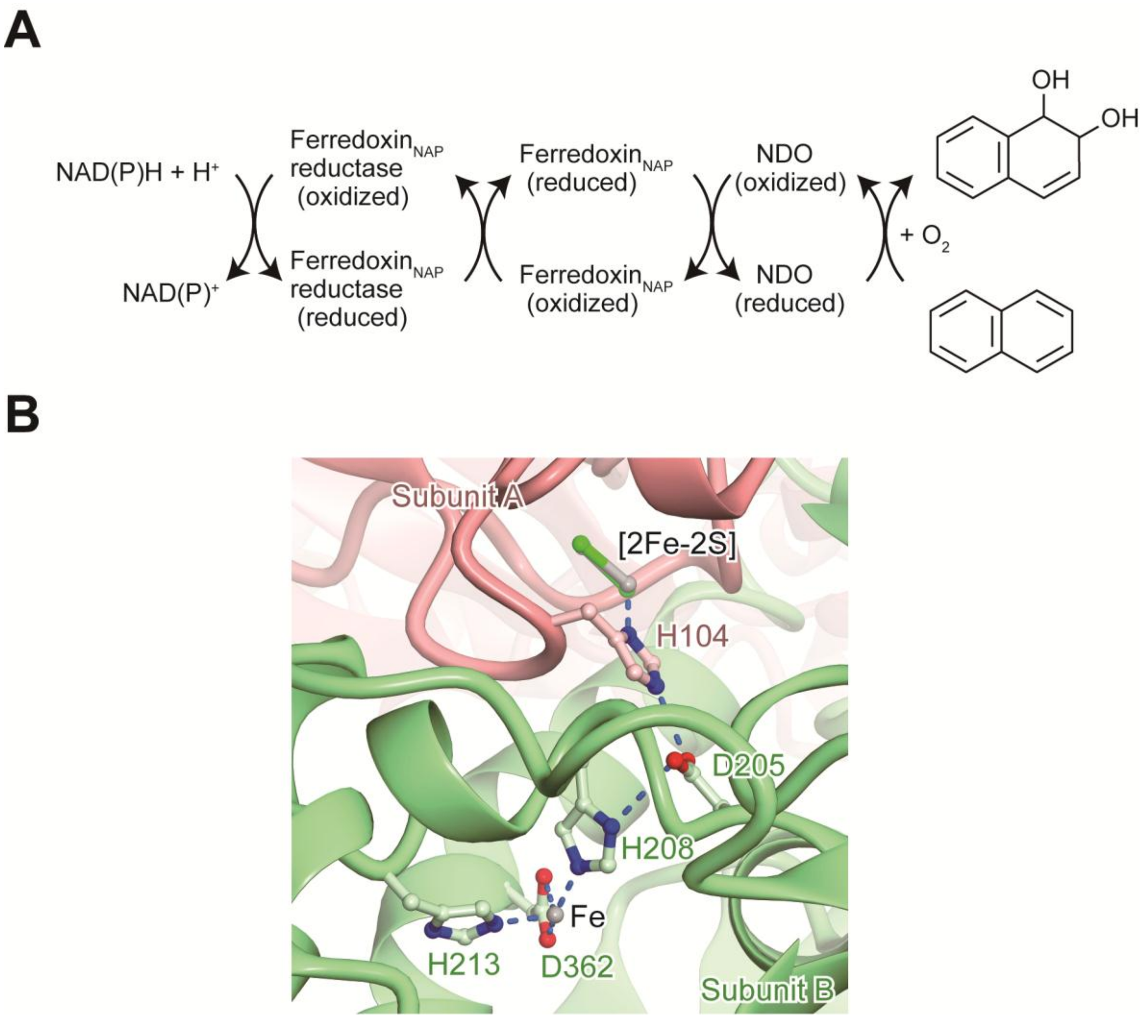


**Figure S6 Hydroxylation of naphthalene by NDO.**

(A) Proposed electron transfer pathway in the naphthalene-hydroxylation reaction (8-11).

(B) Catalytic site of NDO (PDB code: 1NDO) (12). Ribbon model of subunits A and B, colored pink and green, respectively. The side chains and the [2Fe-2S] cluster are depicted as ball-and-stick models. The Fe ion is depicted as a grey sphere.

# Supplementary tables

## Table S1 Data collection and phasing statistics

| Data collection statistics | Native | SeMet (L107M) | | |
|---|---|---|---|---|
| | | Peak | Inflection | Low remote |
| X-ray source | SPring-8 BL32XU | SPring-8 BL41XU | | |
| Wavelength (Å) | 1.0 | 0.97927 | 0.97956 | 0.99529 |
| Space group | *C*2 | *C*2 | | |
| Unit-cell parameters (Å, º) | *a* = 124.4, *b* = 37.0, *c* = 37.6<br>α = γ = 90, β = 101.2 | *a* = 124.8, *b* = 37.2, *c* = 37.7<br>α = γ = 90, β = 101.7 | | |
| Resolution (Å) | 50-1.45<br>(1.48-1.45) | 50-1.95<br>(1.98-1.95) | 50-1.95<br>(1.98-1.95) | 50-1.98<br>(2.01-1.98) |
| Unique reflections | 29,327 (1,400) | 12,484 (594) | 12,318 (482) | 11,744 (487) |
| Redundancy | 5.8 (4.0) | 10.6 (7.6) | 5.3 (3.7) | 5.1 (3.7) |
| Completeness (%) | 97.5 (93.7) | 99.5 (96.6) | 97.3 (75.9) | 97.6 (84.0) |
| $I/\sigma(I)$ | 45.4 (3.3) | 35.7 (7.4) | 24.1 (4.7) | 19.9 (4.0) |
| *R*sym | 0.057 (0.301) | 0.116 (0.301) | 0.106 (0.296) | 0.115 (0.316) |
| **Phasing statistics** | | | | |
| No. of Se sites | | 2 | | |
| Phasing power | | | | |
| Iso (cen./acen.) | | - | 0.896/1.090 | 0.780/0.870 |
| Ano | | 0.283 | 1.198 | 0.085 |
| $R_{cullis}$ | | | | |
| Iso (cen./acen.) | | - | 0.708/0.682 | 0.736/0.743 |
| Ano | | 0.979 | 0.764 | 0.999 |
| Mean FOM | | | | |
| Cen./Acen. | | 0.43905/0.49848 | | |

The numbers in parentheses are for the last shell.

**Table S2 Refinement statistics of native YfcM**

| Refinement statistics | Native |
|---|---|
| Resolution (Å) | 36.917–1.448 |
| No. of reflections (all/test) | 29326/1488 |
| $R_{work}/R_{free}$ | 0.1478/0.1745 |
| No. of atoms | |
| Protein | 1118 |
| Metal | 1 |
| Water | 77 |
| RMSD of | |
| Bond length (Å) | 0.005 |
| Bond angle (°) | 0.953 |
| Average *B* factor (Å) | |
| Protein | 23.8 |
| Metal | 24.4 |
| Water | 33.0 |
| Ramachandran plot | |
| Favored region (%) | 97.0 |
| Outliers (%) | 0.0 |

The numbers in parentheses are for the last shell.

**Table S3 Detection of YfcM-bound Fe ion by atomic absorption spectrometry**

| | Apo | Fe-bound |
|---|---|---|
| Fe (ppm) | < 0.2 | 0.8 |
| [Fe] (μM) | < 3.6 | 14.3 |
| YfcM (mg/ml) | 1.6 | 0.86 |
| [YfcM] (μM) | 72.1 | 38.7 |
| [Fe]/[YfcM] | < 0.05 | 0.37 |

**Table S4 Data collection and refinement statistics of Co(II)-bound YfcM**

| **Data collection statistics** | **Co(II)-bound** |
|---|---|
| X-ray source | SPring-8 BL41XU |
| Wavelength (Å) | 1.60490 |
| Space group | *C*2 |
| Unit-cell parameters (Å, º) | $a$ = 124.3, $b$ = 36.9, $c$ = 37.5<br>$\alpha = \gamma = 90$, $\beta = 101.3$ |
| Resolution (Å) | 50-1.96 (1.99-1.96) |
| Unique reflections | 12,114 (559) |
| Redundancy | 6.3 (5.4) |
| Completeness (%) | 99.1 (94.4) |
| $I/\sigma(I)$ | 49.1 (11.0) |
| *R*sym | 0.061 (0.153) |
| **Refinement statistics** | |
| Resolution (Å) | 36.820-1.960 |
| No. of reflections (all/test) | 23162/1162 |
| $R_{work}/R_{free}$ | 0.1700/0.2067 |
| No. of atoms | |
| Protein | 1128 |
| Metal | 1 |
| Water | 73 |
| RMSD of | |
| Bond length (Å) | 0.008 |
| Bond angle (°) | 0.982 |
| Average *B* factor (Å) | |
| Protein | 24.0 |
| Metal | 30.1 |
| Water | 31.6 |
| Ramachandran plot | |
| Favored region (%) | 97.0 |
| Outliers (%) | 0.74 |

The numbers in parentheses are for the last shell.

**Table S5 Co(II)-coordination manner by YfcM**

| Ligand | Atom | Distance from the Co(II) ion (Å) |
|---|---|---|
| His59 | NE2 | 2.0 |
| His63 | NE2 | 1.9 |
| Glu98 | OE1 | 2.3 |
| Glu98 | OE2 | 2.5 |
| $H_2O$ | O | 2.2 |
| $H_2O$ | O | 2.2 |

## Table S6 m/z values of precursor and transition ions used to discriminate between hydroxylated and non-hydroxylated (*R*)-β-lysyl EF-P

| Peptide | Precursor ion +5 (m/z) | Precursor ion +4 (m/z) | Precursor ion +3 (m/z) *[1] | Transition (y20 +2) | Retention time |
|---|---|---|---|---|---|
| IMLDGEPYAVEASEFVKPGK$^{\beta\text{-lys}}$ GQAFAR | 588.51 | 735.38 | 980.17 | 980.17 --> 1140.67 | 48.89 ±0.3 |
| I(OxM)LDGEPYAVEASEFVKPGK$^{\beta\text{-lys}}$ GQAFAR | 591.71 | 739.39 | 985.51 | 985.51 --> 1140.67 | 47.98±0.3 |
| IMLDGEPYAVEASEFVKPGK$^{OH\ \beta\text{-lys}}$ GQAFAR | 591.71 | 739.39 | 985.51 | 985.51 --> 1148.67 | 48.71±0.31 |
| I(OxM)LDGEPYAVEASEFVKPGK$^{OH\ \beta\text{-lys}}$ GQAFAR | 594.91 | 743.40 | 990.84 | 990.84 --> 1148.67 | 47.97±0.41 |

*[1] only the +3 precursor ion was used for extracted ion chromatogram quantifications

## Table S7 Hydroxylation of Lys34 of β-lysylated EF-P by WT and variants of YfcM

| | Hydroxylated EF-P (%) | | | Hydroxylated EF-P (μM) | | | | |
|---|---|---|---|---|---|---|---|---|
| Sample | A | B | C | A | B | C | Avg conc (μM)*[1] | Std Dev*[2] |
| No protein | 1.52 | 1.85 | 1.13 | 0.08 | 0.09 | 0.06 | 0.07 | 0.02 |
| WT | 5.48 | 7.12 | 5.45 | 0.27 | 0.36 | 0.27 | 0.30 | 0.05 |
| H59A | 1.64 | 1.59 | 0.79 | 0.08 | 0.08 | 0.04 | 0.07 | 0.02 |
| H63A | 1.81 | 1.11 | 1.07 | 0.09 | 0.06 | 0.05 | 0.07 | 0.02 |
| E98A | 1.66 | 0.81 | 1.00 | 0.08 | 0.04 | 0.05 | 0.06 | 0.02 |
| +2,2′-Bipyridyl | 1.88 | 2.14 | 2.04 | 0.09 | 0.11 | 0.10 | 0.10 | 0.01 |
| D105A | 1.67 | 1.89 | 2.46 | 0.08 | 0.09 | 0.12 | 0.10 | 0.02 |
| (Gly-Ser)$_5$ loop | 0.46 | 0.51 | 0.68 | 0.02 | 0.03 | 0.03 | 0.03 | 0.01 |
| Ala$_{11}$ loop | 1.53 | 0.78 | 0.89 | 0.08 | 0.04 | 0.04 | 0.05 | 0.02 |
| -NADPH | 3.32 | 3.39 | 3.01 | 0.17 | 0.17 | 0.15 | 0.16 | 0.01 |

*[1]Average concentration

*[2]Standard deviation

## References


1. Chambers, M.C., Maclean, B., Burke, R., Amodei, D., Ruderman, D.L., Neumann, S., Gatto, L., Fischer, B., Pratt, B., Egertson, J. *et al.* (2012) A cross-platform toolkit for mass spectrometry and proteomics. *Nat. Biotechnol.*, **30**, 918-920.
2. Xu, H. and Freitas, M.A. (2009) Automated diagnosis of LC-MS/MS performance. *Bioinformatics*, **25**, 1341-1343.
3. Peterson, A.C., Russell, J.D., Bailey, D.J., Westphall, M.S. and Coon, J.J. (2012) Parallel reaction monitoring for high resolution and high mass accuracy quantitative, targeted proteomics. *Mol. Cell. Proteomics*, **11**, 1475-1488.
4. Yanagisawa, T., Sumida, T., Ishii, R., Takemoto, C. and Yokoyama, S. (2010) A paralog of lysyl-tRNA synthetase aminoacylates a conserved lysine residue in translation elongation factor P. *Nat. Struct. Mol. Biol.*, **17**, 1136-1143.
5. Doerfel, L.K. and Rodnina, M.V. (2013) Elongation factor P: Function and effects on bacterial fitness. *Biopolymers*, **99**, 837-845.
6. Peil, L., Starosta, A.L., Virumae, K., Atkinson, G.C., Tenson, T., Remme, J. and Wilson, D.N. (2012) Lys34 of translation elongation factor EF-P is hydroxylated by YfcM. *Nat. Chem. Biol.*, **8**, 695-697.
7. Pei, J., Kim, B.H. and Grishin, N.V. (2008) PROMALS3D: a tool for multiple protein sequence and structure alignments. *Nucleic Acids Res.*, **36**, 2295-2300.
8. Haigler, B.E. and Gibson, D.T. (1990) Purification and properties of ferredoxinNAP, a component of naphthalene dioxygenase from Pseudomonas sp. strain NCIB 9816. *J. Bacteriol.*, **172**, 465-468.
9. Haigler, B.E. and Gibson, D.T. (1990) Purification and properties of NADH-ferredoxinNAP reductase, a component of naphthalene dioxygenase from Pseudomonas sp. strain NCIB 9816. *J. Bacteriol.*, **172**, 457-464.
10. Resnick, S.M., Lee, K. and Gibson, D.T. (1996) Diverse reactions catalyzed by naphthalene dioxygenase from Pseudomonas sp strain NCIB 9816. *J. Ind. Microbiol.*, **17**, 438-457.
11. Parales, R.E., Lee, K., Resnick, S.M., Jiang, H.Y., Lessner, D.J. and Gibson, D.T. (2000) Substrate specificity of naphthalene dioxygenase: Effect of specific amino acids at the active site of the enzyme. *J. Bacteriol.*, **182**, 1641-1649.
12. Kauppi, B., Lee, K., Carredano, E., Parales, R.E., Gibson, D.T., Eklund, H. and Ramaswamy, S. (1998) Structure of an aromatic-ring-hydroxylating dioxygenase-naphthalene 1,2-dioxygenase. *Structure*, **6**, 571-586.
